# Education case report: CAMPEP Medical Physics PhD education program within Engineering

**DOI:** 10.1002/acm2.14037

**Published:** 2023-05-21

**Authors:** Brian W. Pogue, David J. Gladstone, Rongxiao Zhang

**Affiliations:** ^1^ Thayer School of Engineering Dartmouth College Hanover New Hampshire USA; ^2^ Department of Medical Physics University of Wisconsin‐Madison Madison Wisconsin USA; ^3^ Department of Medicine Geisel School of Medicine Hanover New Hampshire USA; ^4^ Radiation Medicine New York Medical College Valhalla New York USA

**Keywords:** Cherenkov, imaging, medical physics, radiotherapy, therapy

## Abstract

Medical physics doctoral programs have large variations in organization, administration and financing. Blending a medical physics stream into an engineering graduate program has advantages of pre‐existing financial and educational infrastructures.

A case study of the accredited program at Dartmouth was carried out, analyzing operational, financial, educational and outcome features.

The support structures provided by each institutional partner were outlined, including engineering school, graduate school, and radiation oncology. The initiatives undertaken by founding faculty were reviewed, along with allocated resources, financial model, and peripheral entrepreneurship activities, each with quantitative outcome metrics.

Currently 14 PhD students are enrolled, supported by 22 faculty across both engineering and clinical departments. The total peer‐reviewed publications are ≈75/year, while the conventional medical physics fraction of this is about 14/year. Following program formation, a significant rise was seen in jointly published papers between engineering and medical physics faculty, up from 5.6 to 13.3 papers/year, with students publishing an average of 11.3/person with 5.7/person as first author. Student support was predominantly via federal grants, with a stable $5.5million/year, using about $610K/year supporting student stipends and tuition. First year funding, recruiting and staff support were via engineering school. Faculty teaching effort was supported by agreement with each home department, and student services were provided by engineering and graduate schools. Student outcomes were exceptional, with high numbers of presentations, awards, and residency placements at research universities.

The lack of financial and student support in medical physics can be mitigated by this hybrid design of blending medical physics doctoral students into an engineering graduate program, providing complementary strengths. Future growth in medical physics programs might consider following this pathway, strengthening research collaborations for clinical physics and engineering faculty, as long as there is vested commitment to teach by the faculty and department leadership.

## 1 INTRODUCTION

Medical physics education has seen a number of significant changes in the last decade, with the most dominant factor being the ABR 2012 requirement for students to have graduated from a Commisson on Accreditation of Medical Physics Education Programs (CAMPEP) accredited graduate program in order to enter the residency match program. This factor caused a number of changes in how graduate programs are administered and operated.^1^ Additionally, there has been a growing opinion that the doctoral degree is a superior preparation for academic medical physics positions,^2^, ^3^ and that training should overtly include research.^4^, ^5^ Yet the funding models available for doctoral medical physics education programs are less clear than the master's programs. This is because doctoral candidates traditionally have full tuition and stipend support for approximately 5 years, while most master's programs are self‐funded by the students. Yet, this comes at a time when there is a decreasing number of medical physicists who have extramural funding.^6^ Additionally, the majority of medical physics programs do not have their own departmental status and so they cannot control their own budget, and most are embedded into clinical departments with decreasing focus on research time. These shifts have limited the potential to find funding for medical physics doctoral programs, while at the same time there has been increased demand for creation of these training opportunities. These trends present a problematic path forward for sustainable development of models for medical physics doctoral education. This is the rationale for the program analyzed here, where a solution was sought for continued growth of educational opopportunities for academic medical physics, that woudl be built from intersecting areas of research strength and financial capability.

Several institutions have tried to design doctoral programs that cross departments and schools within the university, as a way to blend a clinically driven degree with a conventional research‐based program, searching for institutional support mechanisms that will make the program viable. The most dominant of these has been the blending of medical physics into traditional physics departments or into biomedical engineering (BME) departments. The growth within engineering schools is seeing more activity given the conceptual match to applied or device/software‐driven research. The focus of this study was a case study of the system set up at Dartmouth, which blended the substantial student and grant resources of the Engineering School with the clinical resources of the Dartmouth Health Medical Center and the faculty from the Geisel School of Medicine in the Division of Radiation Oncology.

## 2 METHODS AND APPROACH

The creation of a new medical physics graduate program with CAMPEP accreditation was completed at Dartmouth in the 2015 academic year with accreditation in 2016, bootstrapping it through the use of existing institutional resources, including a mix of engineering school, graduate school and radiation oncology support mechanisms. The rationale and goal of the program was to develop a research‐focused doctoral degree pathway for medical physics training that would be synergistic with the research and teaching of the faculty and provide an accredited pathway for future graduates. A retrospective analysis of the development and the outcomes is beneficial to appreciate the components that made the system work, and review how medical physics research and education can co‐exist embedded within a BME administrative structure.

The formation of the program was initiated by the founding faculty, partially in response to the requirements placed by CAMPEP, and partially because it was a good opportunity institutionally and geographically for a new medical physics training program in New England.^7^ The founding faculty were a blend from two areas, with BME faculty who have a primarily teaching and research focused mandate, and the clinical physics faculty who have clinical physics focus, with direct access and capabilities to utilize the imaging and therapy systems for research. The two groups had developed cross adjunct appointments and were already converging on a higher level of translational‐research interaction, with support from the Section of Radiation Oncology and the Dartmouth Cancer Center.

The program exists with 22 elected faculty, a triumvirate of faculty directors coordinating all aspects of the CAMPEP program, with one each from (i) Engineering, (ii) Radiation Oncology and (iii) Radiology, and draws individual faculty from a range of other medical school departments including surgery, orthopedics and medicine, but with the dominant fraction being from the engineering school. The program was CAMPEP accredited for just the PhD degree, and those entering are admitted to the engineering school PhD program, with a focus area of BME and medical physics. The Medical Physics accreditation is awarded through the graduate school though, directly by communication from the *Med Phys* faculty director to the Graduate School registrar, upon completion of the required CAMPEP coursework. The *Med Phys* program is approximately 40%–50% of the BME faculty and student body. But the engineering school does not have a BME department, rather it is an area of emphasis for students who all graduate with an engineering sciences designation. Of the seven required CAMPEP courses, three are taught through the engineering school (Medical Imaging, Radiation Transport, and Quantitative Human Physiology) while the remaining four are taught as Graduate School classes (Radiobiology, Radiation Safety and Dosimetry, Radiation Therapy Physics, and Practicum in Medical Physics). All courses comply with the AAPM Report 197s and CAMPEP guidelines.^8^ Small changes were made to each course to ensure that the content coverage matched the CAMPEP requirements, the details of which are too much to include here. Briefly, the Medical Imaging systems course was developed to follow the comprehensive coverage overview in the standard Bushberg textbook.^9^ The radiation transport class was augmented with detection and instrumentation discussions. The physiology class was not able to incorporate cross sectional anatomy directly due to time constraints, so that supplementary sources were found for instruction of this for all medical physics students, and just recently a dedicated course was developed on this topic. Each course is taught every 2 years, so that it is possible to complete them all within a 2‐year cycle of any student.

The major components that drove the design of the program were:

1. engineering school support mechanisms in faculty teaching, research foci, first year student support, staff support for student life, Bio Safety Level 2 (BSL2) BME research facilities at the medical center, and pre‐ and post‐ award grant management;
2. radiation oncology expertise and support for access to clinical resources after hours, medical physicist teaching support, synergy with physician residents;
3. graduate school support for creating interdisciplinary pathways, support for the CAMPEP accreditation management, and student services supports,
4. faculty/department willingness to teach/support CAMPEP classes prior to funding, subsequent funding of course teaching upon achieving critical mass,
5. bootstrapping processes prior to critical mass via the engineering school, building courses around pre‐existing BME curricula;
6. supporting all students on extramural research funding with no request to change funding pathways;
7. strong pre‐existing research resources including cancer center shared services, pilot funds, student grant writing training, robust faculty grant support, as well as peripheral entrepreneurial startup support.

Each of these areas are touched on in the following paragraphs within the Methods section.

### 2.1 Engineering school

The program was created by leveraging faculty and courses within the Thayer School of Engineering that pre‐existed to serve BME graduate students in the areas of imaging and therapy systems. Three core classes already existed prior to the program formation, including Medical Imaging, Radiation Transport, and Quantitative Human Physiology, as well as a robust ethics training program, and scientific professional development training. To achieve CAMPEP requirements, four additional courses were required to be organized and taught, including Radiation Therapy Physics, Radiation Safety and Protection, Radiobiology and a Medical Physics Practicum. These latter courses were taught by a mix of faculty at the medical school and engineering school who were supervising PhD students already, and the courses were staffed by volunteer teaching for several years. These additional courses were all taught just once every 2 years. All student facing organization of the program is supplied by the engineering school, including admissions, orientation, degree management, stipend management, and health insurance, as they are admitted by the engineering school as PhD students in the area of BME. Tenure track faculty employed by the engineering school have expectation for teaching three full courses per year, and inclusion of these medical physics courses in that expectation was possible. Additionally, research track and adjunct faculty are commonly employed to teach full graduate classes when needed, and this was eventually developed to cover most of the classes. Financial support for teaching the non‐engineering classes has not been resolved yet, but continues to be taught by medical school faculty.

Perhaps one of the most synergistic support mechanisms was that the BME program purchased a large research laboratory space (8000 sq. ft.) within the medical center, at the same time that the Medical Physics program was founded. This facility led to the relocation of a dozen BME faculty and their students and staff into a permanent location within the medical center. Here applied and translational biomedical research could be done, with BSL2 facilities and direct access to the cancer center shared services for imaging and therapy. Additionally, through a combination of institutional and extramural funding, an Advanced Imaging facility was created, providing large animal and human imaging and interventional study space. Prior to all of this, there had always been extensive involvement of BME in the medical center, with strong links to surgery, neurosurgery, radiology and radiation oncology, but with just less dedicated spaces. These infrastructures were created through extensive research funding from the National Institutes of Health (NIH), and the engineering school developed a strong and very robust pre‐ and post‐award grant management system that is heavily utilized. Subsequently the engineering school invested in clinical trial support personnel with two full time staff, to manage IRB and FDA applications for the researchers. All these investments in space and people was highly synergistic with gaining sufficient extramural funding to support the medical physics students.

### 2.2 Radiation oncology

Radiation Oncology is a division within the Department of Medicine at Dartmouth Health, and has had a robust internal clinical medical physics program, supportive of the research and teaching mission of the university. There was a prior history of supporting access to clinical resources such as CT, treatment planning, and linear accelerators for animal and veterinary radiotherapy research. Most imaging and therapy works are done after clinical hours, and a management system was set up as part of the Comprehensive Cancer Center's institutional Shared Resources. Subsequent to the medical physics education program, a radiation oncology residency program was founded, and the dual education missions of PhD students and medical residents was synergistic. There was a 2 course overlap between these two groups of trainees in education/teaching of radiation biology and therapy physics, as well as in development of joint research projects.

The medical physics faculty employed at the medical center hold academic appointments at the Geisel School of Medicine at Dartmouth and are adjunct faculty in the Thayer School of Engineering as needed for participation in the PhD program. Medical Physics faculty in radiation oncology were allotted 20% time for academic pursuit and it was understood that clinical resources such as the linear accelerators, scanners, and so forth were available for their academic needs. Consideration of these resources as part of the shared resource of the National Cancer Institute (NCI) funded cancer center core grant enabled radiation oncology to contribute to the shared resource providing consulting, imaging, and radiation delivery to investigators throughout the academic system while simultaneously providing opportunity for academic growth by participation in ongoing research endeavors. This structure created an unusually rich shared resource to investigators while simultaneously providing significant opportunities for participation by the clinical faculty in the academic mission. The medical physics education program was therefore a natural extension of this participation with clinical medical school faculty serving as advisors and thesis committee members for enrolled students.

### 2.3 Graduate school

The Dartmouth Guarini School of Graduate Education and Research provided institutional support for creation of interdisciplinary pathways in graduate education. There was a pre‐existing history of testing out programs prior to their being considered for their own graduate program, and the concept of establishing a CAMPEP accreditation pathway for the existing PhD students in engineering science was approved upon request, provided that minimal financial support was needed. The management of the granting of all PhD degrees is through the graduate school, and so additional awarding of the CAMPEP accreditation on each student transcript is managed through this school as well through communication with the medical physics program director.

### 2.4 Initiative by faculty and departments

Perhaps the most important step in establishing the CAMPEP pathway is the commitment to teach the seven required classes, and the process of teaching this prior to accreditation is a challenging bootstrapping activity, whereby existing resources were used without input of funding. Several of the courses were initially taught as reading courses, or independent study, prior to accreditation, and students followed the alternate pathway to achieve CAMPEP accreditation through completing independent review of each course. The willingness of the participating faculty to teach the courses in the absence of expected compensation was critical to getting the program running, and the support of their employing departments to allow this was also critical. The university departments at the medical school have a strong focus on education and research, and so this was possible without the need of specific recruitment of faculty for this program.

#### 2.4.1 Bootstrapping and embedding medical physics with engineering

Starting up a new graduate program with only 1–2 students per year is not an ideal environment in which students can thrive, and so nesting these students in with BME was one of the critical designs. The students enter the program as BME major PhD students, and simply choose the CAMPEP medical physics courses as 7 of their required 8+ courses in BME. Most students are identified on the Medical Physics track during their application and admissions process with the offer of joining the program. Although some students subsequently discover medical physics after matriculation into BME and can be admitted to the medical physics stream based upon application and successful review of their materials by the medical physics directors. The admissions requirements are identical for BME students and engineering medical physics stream students. The requirements in engineering are flexible, allowing for up to half of courses to be taken outside of engineering, and so those courses that were set up as Medical Physics courses counted as outside electives. The three engineering branded courses, Medical Imaging, Radiation Transport and Quantitative Human Physiology counted for the courses inside of engineering. It is common for students to take more than the minimum eight required courses, and there is a recommendation for courses in three categories, including at least two applied math, at least three breadth courses, and at least three depth courses. Additionally, by embedding the program in the engineering school there is a high economy of scale, in that there was no need for a separate staffing of admissions, registrar, student deans, career services, and student life people. Additionally, all required elements for the engineering PhD applied equally well to the Medical Physics students, such as attendance at weekly seminars, mandatory ethics training, Teaching Assistantships, stipend and health insurance support, professional training in grant writing, and extramural opportunities.

#### 2.4.2 Financial model

The engineering school supports all admitted students for the first nine months on a full stipend independent of their research advisor, and then there is an expectation of the advisor financially supporting the students fully after that time, with stipend, health insurance and tuition support to the engineering school, totaling to the NIH student support level. The Medical Physics program followed the engineering school funding model and did not require any additional financial support upon startup, simply adding to the applicant and matriculant pool of the BME pathway. It is generally viewed that the Medical Physics stream of the BME PhD program is a positive competitive attractant to the engineering applicant PhD pool. The fee for CAMPEP accreditation review was funded by the engineering school.

Other than teaching support for the engineering branded classes, the Medical Physics program receiveed no support for its students, and they are largely managed and served by the engineering school for all their other needs. The faculty run their grants through their home departments, largely engineering, with some in medicine or surgery. Those faculty primarily appointed at the Dartmouth Health work in a different financial system than those at Dartmouth College, and so transfers of funding between the engineering school and the medical center are based upon faculty involvement although student support were managed through these transfers. Funding transfer between those faculty at the medical school or cancer center to the engineering school is not needed, as they are all within the Dartmouth institution, and have joint access to accounts.

#### 2.4.3 Research and entrepreneurship

The program was formed from a robust pre‐existing research and entrepreneurship culture in the faculty that was synergistic with medical physics training activities. Additionally, the substantial infrastructure of the Dartmouth Cancer Center with research resources and pilot grant funds was essential. There was growth in the number of graduating students and increased diversity of grants funded that expanded the research topics worked on by students. Peripheral entrepreneurial startups in the space of medical devices for imaging and therapy was highly synergistic, supporting faculty, researchers, students and graduates of the program through SBIR/STTR subcontracts and other capital gained through the companies where research development was needed.

The sections listed above describe areas that were thought to be critical inputs to creation of the program, however not all of these topics can be objectively assessed for their value. So, in the analysis, the results presented here focus on those areas that can be quantified or tabulated in a meaningful manner. This included: (1) summary student data; (2) faculty publications; (3) funding; (4) CAMPEP program summary statistics.

## 3 RESULTS

### 3.1 Student outcome data

All individual data found that would have relevance to assessing the quality of the individual students is tabulated in Table 1. Table 2 reports on summary outcome data for the entire student body in numbers for the 10 students who have completed the program, quantifying first author publications (*n* = 57), all peer reviewed papers (*n* = 117), conference papers (*n* = 37), oral presentations (*n* = 100), poster presentations (*n* = 61), patents (*n* = 8), national awards (*n* = 14) and local chapter awards (*n* = 7).

**TABLE 1:** Individual graduated student's data (*n* = 10) for their entire PhD output, from the CAMPEP program were compiled and outcome metrics of their accomplishments that were directly attributable to their graduate study were analyzed and summarized here. Numbers listed are mean values and total range in parentheses. All students went on to a residency.

# Peer reviewed papers	11.3 (6–15)
# first author Peer Reviewed papers	5.7 (2–11)
# conference papers	3.7 (0–8)
# oral presentations	10.0 (7–18)
# poster presentations	6.1 (2–11)
# patents	0.8 (0–2)
# National awards	1.4 (0–5)
Early investigator symposium	5 of 10
Independent funding	3 of 10
# women for gender diversity	3 of 10
# in 3‐year research residency	4 of 10
Match and non‐match residency	5 and 5

**TABLE 2:** Cumulative academic productivity for the students (*n* = 10) graduating from the program.

Peer reviewed first author	Peer reaviewed	Conference papers	Oral presentations	Poster presentations	Patents	National Awards	Chapter Awards
57	113	37	100	61	8	14	7

### 3.2 Faculty data

**FIGURE 1:**
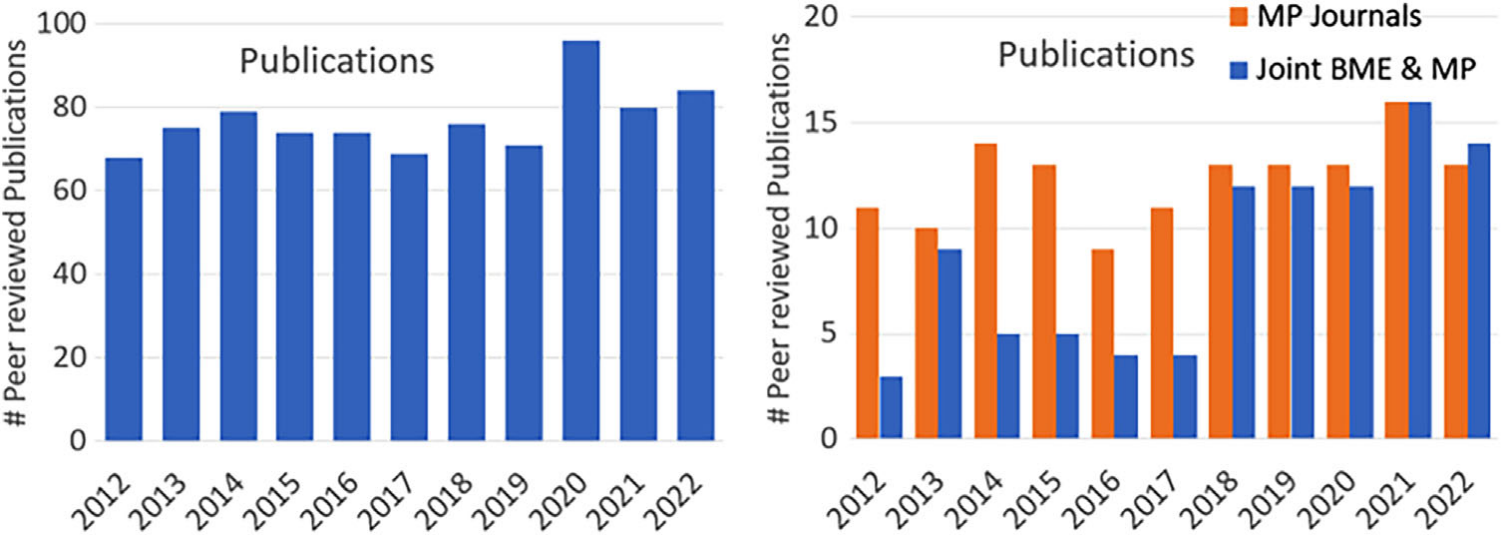
Complete publication data (a) was compiled from PubMed search on the faculty names in the program, for 3 years before program formation through to 2022. Subsequent refinement of this showed those publications in medical physics related journals (b) and those that were jointly published between BME and MP faculty as co‐authors.

Searches on Medical Physics faculty publications (PubMed) and NIH funding (NIH reporter) where the program faculty names were searched cross listed with Dartmouth or Dartmouth‐Hitchcock in the institution was completed, and the cumulative output trend is shown in Figure 1. The publications for 2022 are likely not completed yet, but otherwise there is a stable trend with a notable increase in 2020 and 2021. A separation of those publications in Medical Physics and the *International Journal of Radiation Oncology, Biology, Physics* shows that only a minority of publications wind up in the medical physics field, about 15%, and there is a small but growing trend to increasing publications in *IJROBP*, suggesting higher impact papers.

### 3.3 Funding data

**FIGURE 2:**
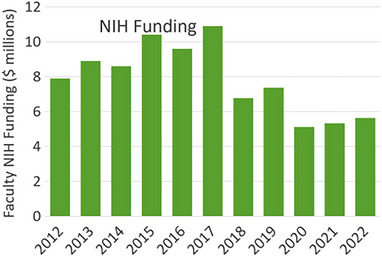
DHHS funding made up the predominant fraction of support for faculty in the program, and a search from NIH Reporter showed the annual total funding numbers for those faculty involved. There was insigificant funding from other government sources during this period. DHHS, Department of Health and Human Services; NIH, National Institutes of Health.

While the funding fluctuated due to faculty changes over time, as shown in Figure 2, currently stable funding in recent years (2020–2022) was approximately $5.5 million/year. The sources of funding were nearly entirely from the Department of Human and Health Services, via the National Institutes of Health (NIH). New faculty additions are evaluated each year by the program leadership, and so funding will fluctuate with membership and individual grant support awards.

Assuming that each student costs faculty support of $50,000/year, except for only summer support in the first year, this is an annual research support cost of approximately $610,000. The department funding covers the first 9 months of all new PhD students, and a reduced tuition is charged to all grants of approximately $14,000/year, as well as health insurance costs. Recruiting, administrative personal, student support, travel funding are all available through the engineering school, which is a major cost savings for the program.

### 3.4 CAMPEP reported data

**FIGURE 3:**
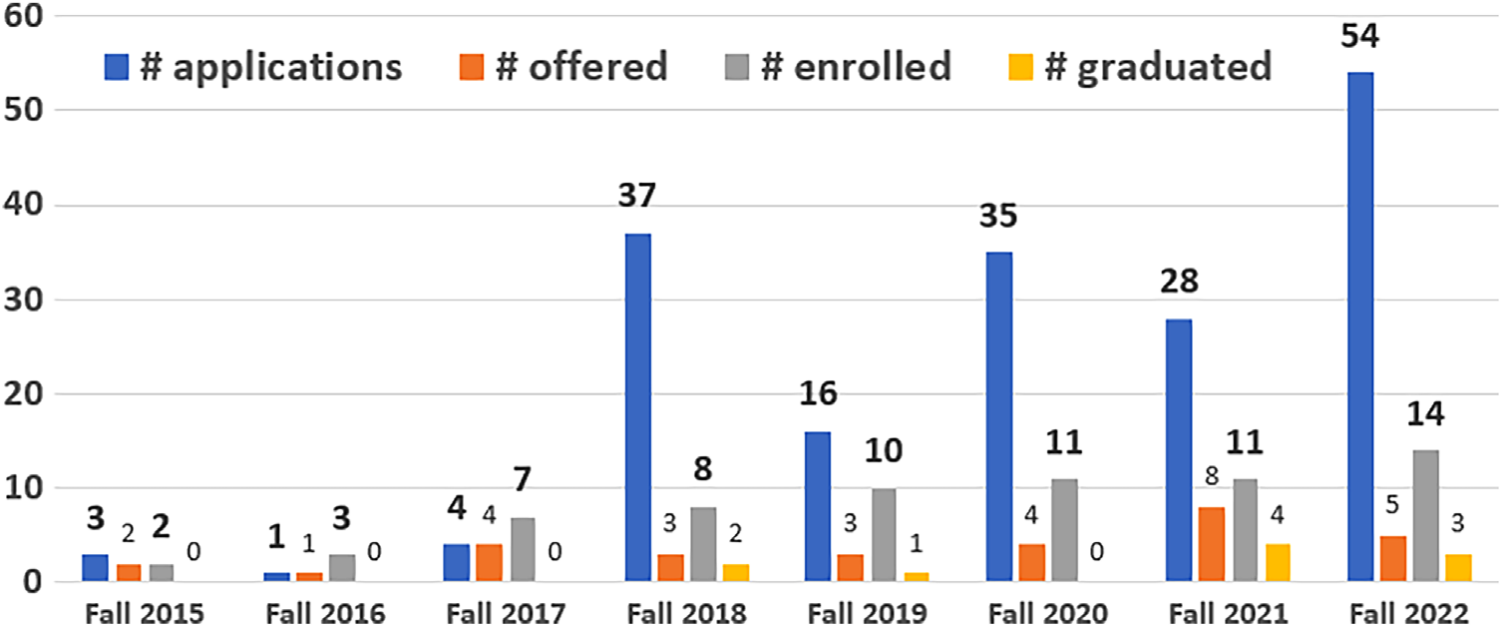
Annual numbers of student applications, offers, entering program, and graduating from the program are shown in the above chart, for the program since inception.

Student numbers involved in the program are tracked by CAMPEP requirements for matriculation, graduation, and those in the program, and are plotted in Figure 3. From the graph it can be seen that the number of students in the program is currently 14, growing consistently over the years.

## 4 DISCUSSION

The results of analysis of the program are a lesson in bootstrapping a program from within the strengths of an academic institution, where leveraging of strategic institutional resources around instruction, education, research, collaboration and entrepreneurship have worked out to support the goals of a CAMPEP accredited program. Some of the more important observations are discussed below in the following sections.

### 4.1 Publications

It is interesting to examine the publications history in Figure 1, which shows that because of the diverse range of faculty involved in the program that only about 15% of the publications resulting during this period wound up in *Medical Physics* journals. This indicates that there was a diversity of science being done, well beyond the traditional areas of medical physics. Indeed, there is a large surgical guidance research program in the group as well as non‐traditional imaging methods, both of which tend to get published in non‐*Medical Physics* journals. Additionally, it is interesting to note that the number of *Medical Physics* publications did not grow dramatically through the period of the CAMPEP program founding, despite the large growth in student numbers. As such, it must be assumed there was a pre‐existing subset of medical physics research prior to the CAMPEP program coming into place. One notable trend was the observation of a slight increase in *IJROBP* papers in that timeframe, indicating an increase in higher impact journal publishing and an increase in projects of interest to the Radiation Oncology community. Additionally, a key indicator of success of the program is the productivity of students in publishing and it is notable that the average number of publications per student for their entire program was 11.3 each with an average of 5.7 as first author. This is a very successful number for a PhD thesis, indicating high scientific output for the individual students within the medical physics program.

The data shown in Figure 1b illustrates a significant shift, where the number of joint publications increased over time. It can be seen that the joint BME and *Med Phys* faculty co‐authored papers grew during this period, going from a 3‐year average of 5.6 in the 2012–2014 time range up to 13.3 in the 2020–2022 time period, a 2.5× increase. As might be expected, the program provided a conduit for collaboration across the faculty disciplines, likely through the joint supervision of PhD students in the program, and intentional growth in more medical physics research funding.

### 4.2 Financial

Notably most of the research support was on the BME end of the faculty, but the desire to do translational research was very high, and the partnership with clinical physics was an ideal conduit. Examining the data in Figure 2, the percent of grant support that existed was higher at the start of the CAMPEP program and then reduced. This was noted to be due to one senior investigator who retired at this point though, and had a substantial research grant with the Biomedical Advanced Research and Development Authority (BARDA). The mean grant funds in the last 3 years was about $5.5 million/year, of which about $610 000/year is spent on student stipends, for a percentage of 11%. So, this number is probably commensurate with the amount of student productivity in publications, where 15% was on the topic of medical physics. A large amount of grant support goes to faculty and staff salaries, supplies and equipment, and there is not a good estimate of what fraction of students were regular BME stream versus Medical Physics stream. The fact that extramural research funding is not necessarily squarely in the field of Medical Physics is reasonable for such a hybrid program of investigators, and this scientific diversity can probably support growth within and around the clinical discipline of Medical Physics.

An underappreciated but critical part of the program financing is the ability to leverage use of all administrative staff and procedures within the engineering school. The admissions office, registrar, student services, career services and research staff were all pre‐existing because of the existing Masters and PhD programs throughout engineering. This also included funding for travel/accommodations for interviews and even travel funding for conference attendance to present research. This considerable financial leveraging by the engineering school was a major factor in the success of the graduate program, and is likely the singular largest factor in the institutional success of this program.

One category of finance that deserves special focus was the support of PhD students in entrepreneurship. The engineering school overtly supports startups that form around the patents of the faculty and students, and one larger effort came in the field of Cherenkov imaging. This company support was leveraged to fund one student per year, which reduced grant support and provided that student with a broader perspective on career and R&D opportunities. Additionally the engineering school has a separate endowed fellowship program for Entrepreneurial Fellows, who apre‐doctoral students and at least one of the candidates was separately funded through that program.

Another area of interest was students funded by extramural support such as the NIH F31 program or other doctoral program funding opportunities. Three of the 10 had independent funding, with 2 on F31 grants and 1 on an F32 training grant. The opportunity to have a largely self‐funded PhD program is an important career boost to those students, and central training on grant writing with a requirement to write one, was part of the expectation in the engineering school PhD program. This can be considered a metric for success in itself.

### 4.3 Program outcomes

The data in Figure 3 shows a growth of the program that had steady interest, albeit with large annual fluctuations in the application numbers, and an admission/matriculation that ramped up over about 5 years to a roughly stable level of 11–14 per year in the program. The average graduate time was about 4.5 years, making this an admission of about 2–3 students per year. Of the 10 students who sought out residencies, all found them, with placement in programs that are highly ranked nationally. Notably 4 of the 10 entered 3‐year research residencies, and 9 of 10 were therapeutic and one went to diagnostic. The geographic placement of the program was ideal, being the only opportunity for students in northern New England, and only one of two CAMPEP PhD programs in New England. From this standpoint, there was a geographic opportunity to provide a key CAMPEP program in a major hub of higher education.

### 4.4 Student outcomes

In terms of measures of success in the student group, only achievements that are directly related to their graduate study were considered. The mean number of first author papers in the group was 5.7 total, and the total number per student was 11.3, both of which are strong numbers for PhD students, although there is no clear way to produce a comparison group for this. This data indicates that overall there was high productivity of the students, and part of this is likely due to their being supported on NIH research grants, which require academic productivity for renewal. The number of conference abstracts for oral and posters, was 160, and average of 16 per student, which was largely at American Association of Physicists in Medicine (AAPM) and the International Society for Optics and Photonics (SPIE) conferences. These numbers are exceptionally high, indicating about four per year per student. Similarly, the number of prizes both local and national, were very high, indicating that the program was very successful in producing valued research. Notably 5 of 10 were within the young investigator programs at either the Spring Clinical AAPM meeting or the AAPM Annual meeting.

Overall, the outcomes performance of the students in publications, prizes and residency placement makes this program look exceptional within the field of medical physics. The program continues to garner national and international interest of prospective students. Due to the students’ productivity and national visibility, acceptance to highly competitive clinical residency programs have been strong as well.

## 5 SUMMARY

When this program is examined objectively by its quantitative data, the joint initiative between BME and medical physics faculty has strategic strengths and benefits that could be valuable for other universities. The individual strengths of the engineering and medical physics faculties are complimentary and together their individual weaknesses were somewhat mitigated. Weaknesses inherent in many clinical medical physics programs are the lack of dedicated research time and extramural funding for student support, while the strengths are direct control of clinical resources, knowledge of practical needs that drive research ideas, and high potential for direct human translation. The weaknesses inherent in most engineering programs is largely the opposite of this, which is the lack of clinical contact and clinical imaging and therapy equipment access. However, the strengths of engineering faculty are the focus on required teaching, extramural research funding, and the existence of administrative infrastructures for graduate education. Taken together the joint program has significant value and synergy, primarily in high productivity and success of the students, and in synergizing joint research/publications across the divide between BME and clinical physics.

The largest challenges to the program have been financial support for instruction of all the CAMPEP classes, as several would not fit into a graduate BME program. However, departmental support for the teaching mission has mitigated this concern, and concerted desire of the individual faculty and faculty leads to support this. Support and partnership with the Graduate School, the Comprehensive Cancer Center and abundant research facilities were all components that made this program work well. Individual student outcomes are universally strong in outcome metrics. All graduates gained a place in research university‐based residency programs, and importantly 4 of the 10 entered 3‐year, research‐based residencies. With the foundation and framework laid out in the CAMPEP Medical Physics Doctoral Program, similar strategies are being utilized to establish a 3‐year medical physics residency program. The program serves a niche opportunity in New England, where there is a high concentration of higher education, and where Medical Physics training with a CAMPEP accreditation has been less available. The program outlined here is a practical institutional design for advancing productive research and training of new medical physicists, and appears to be growing in adoption at several universities.

## AUTHOR CONTRIBUTION

All three authors were involved in data collection, writing and editing of the manuscript.

## CONFLICTS OF INTEREST STATEMENT

The authors have no conflicts to disclose relevant to this article.

## Data Availability

Not applicable.
